# ﻿Diversity of silica-scaled chrysophytes (Chrysophyceae) from Mindanao Island (Philippines) with the description of *Mallomonas pseudopustula* sp. nov.

**DOI:** 10.3897/phytokeys.268.176758

**Published:** 2025-12-19

**Authors:** Evgeniy S. Gusev, Marina E. Ignatenko, Chris Rey Lituanas, Kyla Rose Gumapac, Nikita A. Martynenko, Boris D. Efeykin

**Affiliations:** 1 Severtsov Institute of Ecology and Evolution, Russian Academy of Sciences, Leninsky Prospect 33, 119071 Moscow, Russia Severtsov Institute of Ecology and Evolution, Russian Academy of Sciences Moscow Russia; 2 Institute for Cellular and Intracellular Symbiosis, Orenburg Federal Research Center, Ural Branch, Russian Academy of Sciences, Pionerskaya Street, 11, 460000, Orenburg, Russia Orenburg Federal Research Center, Ural Branch, Russian Academy of Sciences Orenburg Russia; 3 Institute of Biological Sciences, College of Arts and Sciences, Central Mindanao University, Plant Biology Division, Purok 13, Musuan, 8714 Maramag, Bukidnon, Philippines Central Mindanao University Maramag Philippines

**Keywords:** Chrysophyceae, electron microscopy, morphology, new species, siliceous scales, taxonomy

## Abstract

This study presents the first comprehensive investigation of chrysophytes from the Philippines. The flora of silica-scaled chrysophytes from freshwaters of Mindanao Island has been studied by means of scanning and transmission electron microscopy. A total of thirty-six taxa were recorded, including three belonging to *Paraphysomonas*, two to *Spiniferomonas*, one to *Lepidochromonas*, twenty-nine to *Mallomonas* and one to *Synura*. A new species, *Mallomonas pseudopustula***sp. nov.**, was discovered and described. Several unidentified *Mallomonas* taxa were also recorded. The flora of silica-scaled chrysophytes of the Mindanao Island is similar to the flora of neighbouring countries — Vietnam, Indonesia and Malaysia.

## ﻿Introduction

Silica-scaled chrysophytes comprise heterokont algae from the class Chrysophyceae and different evolutionary lineages belonging to three orders: Paraphysomonadales, Chromulinales and Synurales (Škaloud et al. 2013a). These organisms can be colonial or unicellular. They possess a covering of silica scales, bristles and spines, the ultrastructure of which is species-specific. A robust morphological species concept has been established for this group, which is congruent with molecular phylogenetic data (Scoble and Cavalier-Smith 2014; Siver et al. 2015; Pusztai et al. 2023). Modern morphological studies of silica-scaled chrysophytes are based on electron microscopy, which began in the 1950s (Škaloud et al. 2013a). The information accumulated over more than 70 years allows chrysophytes to be considered a model group for biogeographical studies (Kristiansen 2001; Řezáčová and Neustupa 2007; Siver and Lott 2012).

Although silica-scaled chrysophytes in the Tropics have been studied since the 1970s (Compère 1974; Takahashi and Hayakawa 1979), data are currently available from only 23 countries (approximately one-third of all countries with a tropical climate). A diverse tropical flora has been documented from the tropical region (Compère 1974; Takahashi and Hayakawa 1979; Dürrschmidt and Croome 1985; Dürrschmidt and Cronberg 1989; Cronberg 1989; Hansen 1996a; Wujek and Saha 1996; Wei and Yuan 2001; Wei et al. 2014; Gusev et al. 2022b, 2023a). However, data on the diversity of silica-scaled chrysophytes from tropical islands are rather limited. Furthermore, many studies of island floras have been accompanied by descriptions of new species. In total, 57 taxa of silica-scaled chrysophytes were recorded from 14 localities on two small tropical islands, Phu Quoc and Con Son, in Vietnam (Gusev et al. 2022d). From these localities, four new species of the genus *Mallomonas* were described (Gusev et al. 2019a, d, 2023b; Gusev and Kulikovskiy 2020). Dürrschmidt and Cronberg (1989) identified 29 taxa from Sri Lanka, including two new species and one new variety of *Mallomonas* and a new species of *Paraphysomonas*. In Madagascar, 42 taxa have been recorded (Hansen and Kristiansen 1995; Hansen 1996a, b), including three new species of *Mallomonas* and one of *Spiniferomonas*. Wei et al. (2014) reported 36 taxa of silica-scaled chrysophytes from Hainan Island (China) and described a new species of *Paraphysomonas*. During studies of silica-scaled chrysophytes on the island of Java, 19 taxa were identified, including a new species of *Chrysosphaerella* (Kapustin and Gusev 2019). In Indonesian Papua, a new species of *Mallomonas* was described, with 24 other taxa reported (Kapustin et al. 2019; Gusev et al. 2022b). Many taxa described from islands remain endemic to this day.

The numerous islands of the Philippines belong to the Indo-Malaysian-North-Australian phycogeographical region, one of the ten regions defined by Krieger (1932), based on the composition of desmid floras. There were roughly 287 species of microalga documented in the Philippines which were dominated by diatoms (Lituañas 2019; Martinez-Goss 2021; Santos et al. 2023; Argueles 2025). Since then, there were no studies conducted on Chrysophyceae in general and particularly the investigations of silica-scaled chrysophytes in the Philippines.

Algal floristic studies in this poorly-studied region are essential for understanding the biodiversity and biogeography of different algal groups in the Asian tropics. This paper presents the results of a study on the flora of silica-scaled chrysophytes from Mindanao Island, one of the largest islands of the Philippines.

## ﻿Materials and methods

**Figure 1.**
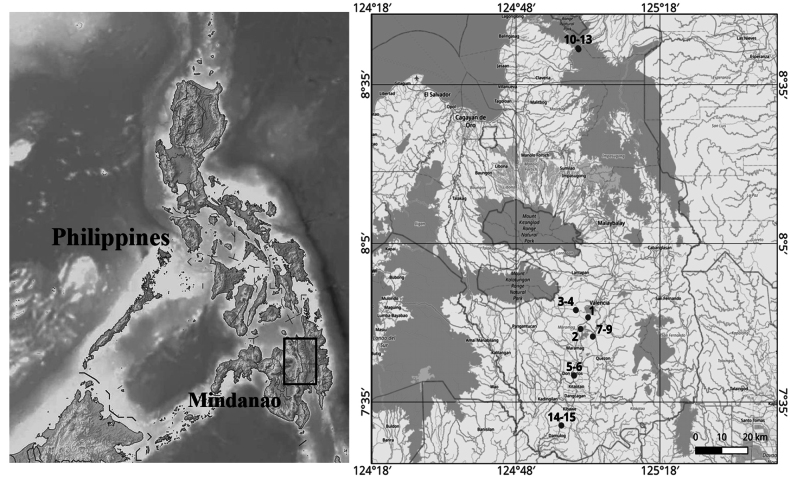
Schematic map of the studied area.

Samples for this study were collected in November–December 2024 and June 2025 on Mindanao Island, Philippines (Table 1, Fig. 1). A total of 13 freshwater localities were studied across three provinces. Mindanao is classified under types II, III and IV in the Modified Coronas Climate Classification System (**MCCS**) used by the Philippine weather agency (**PAGASA**). The study area lies in the Type IV climate zone, which is characterised by rainfall being more evenly distributed throughout the year, without a pronounced dry or wet season.

**Table 1.** Basic characteristics of the studied localities (GPS—coordinates, Cond.—specific conductance, µS/cm, T— temperature, °C).

No.	Localities (year of sampling)	GPS	pH	Cond	T
1	Fullbright Pond, CMU Campus, Musuan, Maramag, Bukidnon (2024)	7°51.410'N, 125°2.998'E	7.3	157	28
2	Pulangi Reservoir, Barangay Bayabason, Maramag, Bukidnon (2025)	7°49.230'N, 125°1.457'E	6.8	156	28
3	Lake Apo, Barangay Guinoyuran, Valencia City (2024)	7°52.776'N, 125°00.487'E	7.6	52	28
4	Lake Apo, Barangay Guinoyuran, Valencia City (2025)	7°52.803'N, 125°00.441'E	8.2	43	28
5	Lake Pinamaloy, Barangay Pinamaloy, Don Carlos, Bukidnon, st. 1 (2025)	7°40.436'N, 125°00.063'E	6.9	53	29
6	Lake Pinamaloy, Barangay Pinamaloy, Don Carlos, Bukidnon, st. 2 (2025)	7°40.421'N, 125°00.141'E	6.6	49	31
7	Opalon Spring, Barangay Butong, Quezon, Bukidnon (2025)	7°47.807'N, 125°03.984'E	7.1	202	27
8	Opalon Stream, Barangay Butong, Quezon, Bukidnon (2025)	7°47.777'N, 125°03.989'E	7.1	200	27
9	Opalon Swamp, Barangay Butong, Quezon, Bukidnon (2025)	7°47.781'N, 125°03.979'E	7.4	155	39
10	Forest waterbody in Barangay Lunotan, Gingoog City, Misamis Oriental (2025)	8°42.054'N, 125°01.044'E	5.5	14	24
11	Pond in Barangay Lunotan, Gingoog City, Misamis Oriental (2025)	8°42.263'N, 125°00.923'E	6.8	19	24
12	Water pool 1 near pond in Barangay Lunotan, Gingoog City, Misamis Oriental (2025)	8°42.263'N, 125°00.923'E	5.2	13	22
13	Water pool 2 near pond in Barangay Lunotan, Gingoog City, Misamis Oriental (2025)	8°42.263'N, 125°00.923'E	6.9	169	30
14	Pawan Resort, Barangay Sampagar, Damulog, Bukidnon (2025)	7°30.964'N, 124°57.460'E	7.4	369	29
15	Abandone Ricefield, Barangay Sampagar, Damulog, Bukidnon (2025)	7°30.976'N, 124°57.421'E	8.3	251	38

Plankton samples were collected from the surface water layer using a plankton net with a mesh size of 20 μm. Fifteen samples were collected and processed. All samples were immediately fixed with Lugol’s solution. Water-specific conductance, pH and temperature were measured in situ using a Hanna HI 9828 multiparameter device (Hanna Instruments, Inc., Ann Arbor, Michigan, USA).

For electron microscopy, an aliquot of each sample was rinsed with deionised water via centrifugation. Drops of the rinsed sample were either air-dried or digested with sulphuric acid and potassium dichromate. For scanning electron microscopy (**SEM**), prepared samples were placed on SEM stubs and sputter-coated with gold using an ion-plasma sputtering system (Quorum Q150R S plus; Quorum Technologies Ltd., London, UK). Observations were carried out with a TESCAN MIRA3 microscope (TESCAN BRNO, s.r.o., Brno, Czechia) at the Gagarin Center for the Identification and Support of Talented Children (Orenburg Oblast, Russia). For transmission electron microscopy (**TEM**), Formvar-coated copper grids (EMS FF200-Cu-50; Electron Microscopy Sciences, Hatfield, PA, USA) were used. TEM observations were conducted with a JEOL JEM-1011 microscope (Papanin Institute for Biology of Inland Waters, Russian Academy of Sciences, Borok, Russia). Twenty-seven scales of the new species were measured to prepare the description.

## ﻿Results

**Figure 2.**
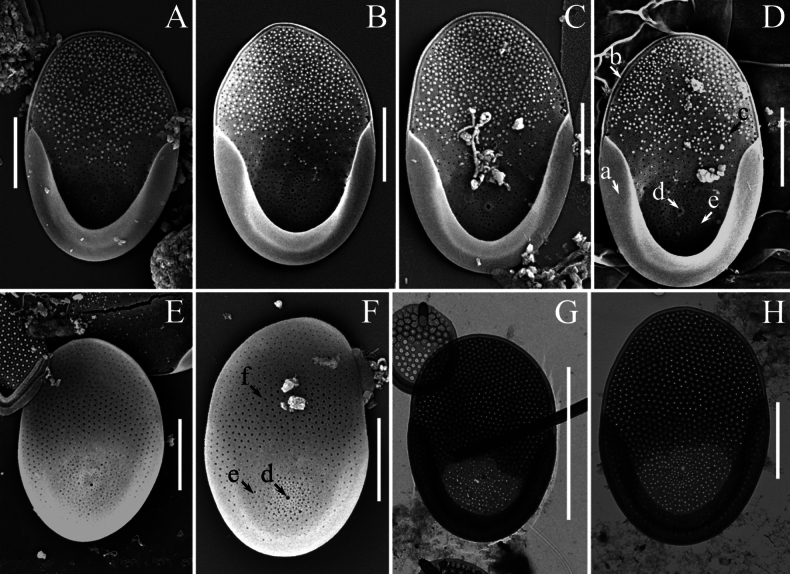
*Mallomonas pseudopustula* sp. nov. **A–D, G, H.** Surface view of the scale; **E, F.** Undersurface view of the scale; Arrows indicate: (**a**) Posterior rim; (**b**) Hyaline belt; (**c**) Thick secondary layer with papillae and pores; (**d**) Large central rimmed pore in the proximal part of the scale surrounded by a “cloud” of small pores; (**e**) Pores in the proximal part of the scale; (**f**) Pores in the distal part of the scale; **A–F.**SEM; **G, H.**TEM. Scale bars: 5 µm (**G**); 2 µm (**A–F, H**).

**Figure 3.**
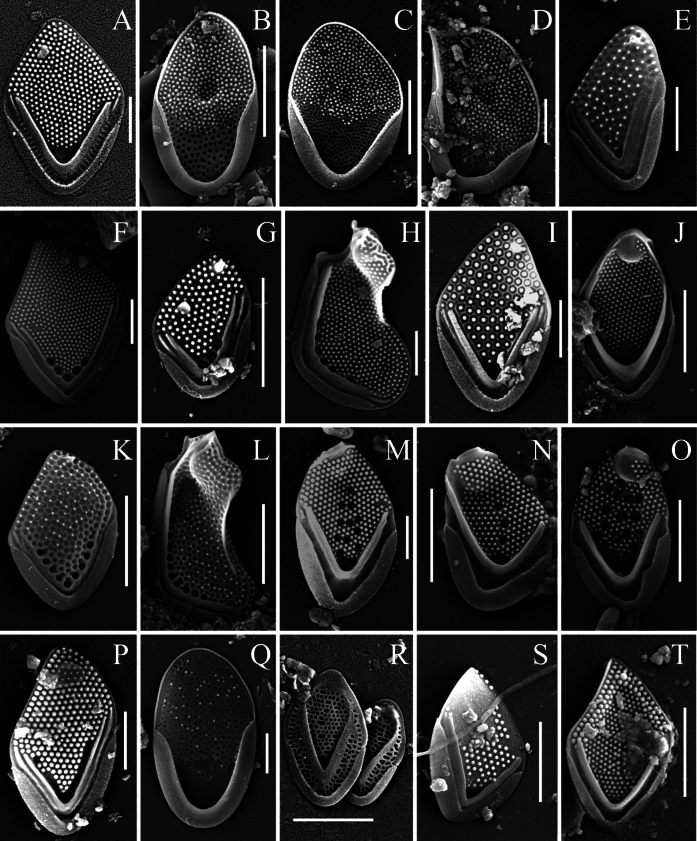
*Mallomonas* taxa from Mindanao Island (Philippines). **A.***Mallomonas cetosa*; **B–D.***Mallomonas ceylanica*, body scales (**B, C**), apical scale (**D**); **E.***Mallomonas favosa*; **F–H.***Mallomonas foveata*, body scale (**F**), rear scale with a small spine (**G**), apical scale (**H**); **I.***Mallomonas fragariformis*; **J.***Mallomonas furtiva*; **K, L.***Mallomonas gemina*, body scale (**K**), apical scale (**L**); **M.**Mallomonas guttata var. guttata; **N.**Mallomonas guttata var. simplex; **O.***Mallomonas* sp. 1; **P.***Mallomonas hippocrepica*; **Q.***Mallomonas kapustinii*; **R.***Mallomonas kenyana*; **S.***Mallomonas kornevae*; **T.***Mallomonas mangofera* apud Dürrschmidt. Scale bars: 2 µm (**B, C, G, J–L, N, O, R–T**); 1 µm (**A, D–F, H, I, M, P, Q**).

**Figure 4.**
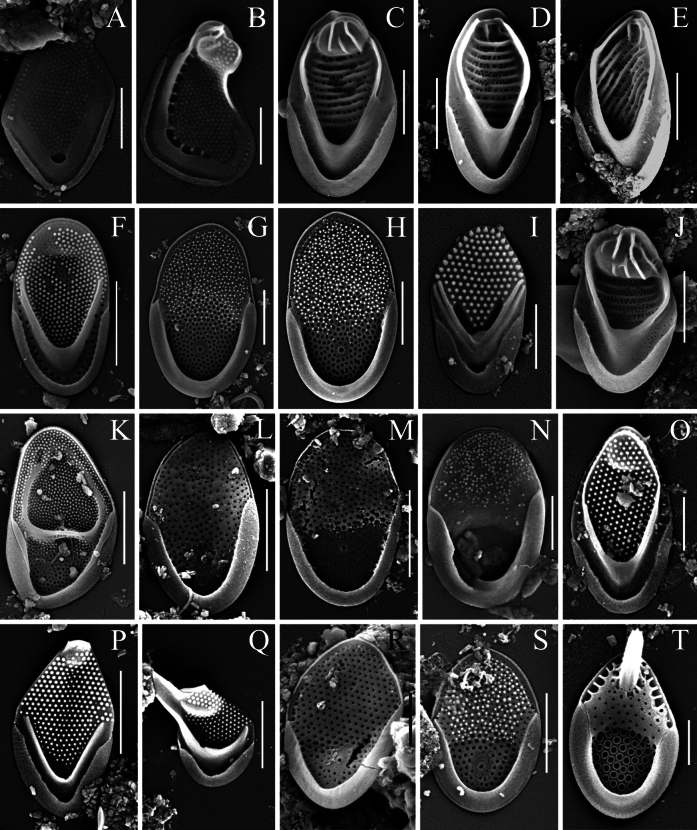
*Mallomonas* and *Synura* taxa from Mindanao Island (Philippines). **A, B.***Mallomonas minuscula*, body scale (**A**), apical scale (**B**); **C–E, J.***Mallomonas morrisonensis*, domed body scale (**C**), domeless body scales (**D, E**), apical scale (**J**); **F.***Mallomonas multisetigera*; **G.***Mallomonas paragrandis*; **H.**Mallomonas aff. paragrandis; **I.***Mallomonas parvula*; **K.***Mallomonas peronoides*; **L**, **M.***Mallomonas pseudomatvienkoae*; **N.***Mallomonas pustula*; **O.***Mallomonas tropica*; **P, Q.***Mallomonas rasilis*, body scale (**P**), apical scale (**Q**); **R.***Mallomonas* sp. 2; **S.***Mallomonas* sp. 3; **T.***Synura longitubularis*. Scale bars: 2 µm (**C–H, J, K–N, P, Q, S**); 1 µm (**A, B, I, O, R, T**).

**Figure 5.**
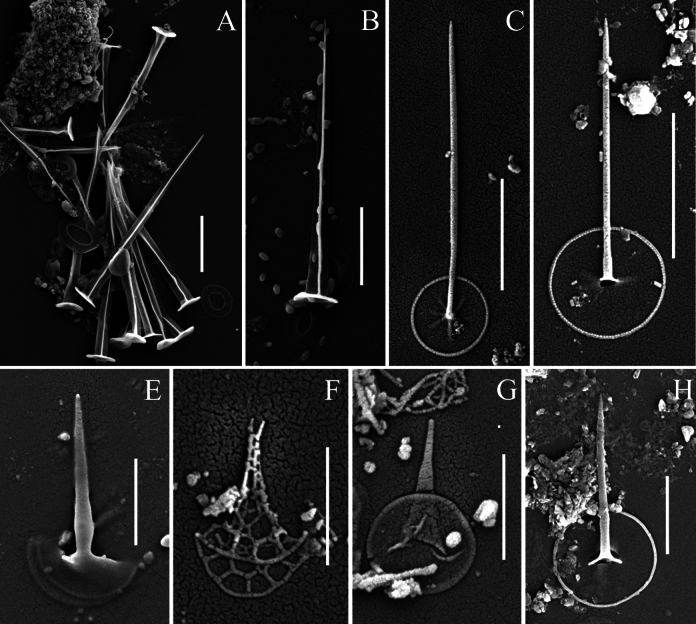
*Spiniferomonas*, *Lepidochromonas* and *Paraphysomonas* taxa from the waterbodies of the Mindanao Island (Philippines). **A, B.***Spiniferomonas trioralis*; **C.**Paraphysomonas uniformis subsp. hemiradia; **D, H.**Paraphysomonas vulgaris subsp. vulgaris; **E.**Spiniferomonas cf. bourrellyi; **F.***Lepidochromonas eiffelii*; **G.**Paraphysomonas cf. corynephora. Scale bars: 2 µm (**A–E, H**); 1 µm (**F**, **G**).

A total of thirty-six taxa of silica-scaled chrysophytes were recorded in the freshwaters of the Philippines (Mindanao Island), including three belonging to *Paraphysomonas* De Saedeleer emend. Scoble & Cavalier-Smith, two to *Spiniferomonas* E. Takahashi, one to *Lepidochromonas* Kristiansen, twenty-nine to *Mallomonas* Perty and one to *Synura* Ehrenberg (Table 2). All identified taxa are new records for the Philippines. Three organisms belonging to the genus *Mallomonas* could only be identified to the genus level and may represent new species, pending further detailed study. Furthermore, a new species of the genus *Mallomonas* was discovered (Fig. 2) and its description is provided below. Several other noteworthy taxa are characterised in the following section (Figs 3–5).

**Table 2.** List of silica-scaled chrysophytes found in the waterbodies of the Mindanao Island (Philippines) (“+” indicates the presence of taxon).

TAXON	1	2	3	4	5	6	7	8	9	10	11	12	13	14	15
*Mallomonas cetosa*														+	
*M. ceylanica*					+			+	+						
*M. favosa*										+					
*M. foveata*					+	+		+	+						
*M. fragariformis*			+										+		
*M. furtiva*					+	+		+	+				+		
*M. gemina*	+							+	+						
M. guttata var. guttata								+	+						+
M. guttata var. simplex				+											
*M. hippocrepica*					+			+							
*M. kapustinii*					+			+	+						
*M. kenyana*		+												+	
*M. kornevae*			+												
*M. mangofera apud* Dürrschmidt, 1983					+	+									
*M. minuscula*									+						
*M. morrisonensis*					+	+					+				
*M. multisetigera*					+	+	+	+	+	+		+			
*M. paragrandis*					+			+	+						
M. aff. paragrandis						+									
*M. parvula*								+	+						
*M. peronoides*	+														
*M. pseudomatvienkoae*					+			+							
*M. pustula*	+														
*M. rasilis*	+		+		+	+									
*M. tropica*								+							
***Mallomonas pseudopustula* sp. nov.**	+				+	+							+		
*Mallomonas* sp. 1					+	+		+			+				
*Mallomonas* sp. 2									+						
*Mallomonas* sp. 3	+														
*Synura longitubularis*	+				+	+			+				+		
Spiniferomonas cf. bourrellyi	+														
*S. trioralis*	+					+									
*Lepidochromonas eiffelii*									+						
Paraphysomonas cf. corynephora	+														
P. uniformis subsp. hemiradia										+					
P. vulgaris subsp. vulgaris	+	+	+	+		+		+	+		+				

### ﻿ Mallomonas pseudopustula

Taxon classificationPlantaeSynuralesMallomonadaceae

E.S. Gusev, Ignatenko, Lituanas, Gumapac & Martynenko sp. nov.

0A570191-A98B-5A0C-96D2-A05571B2D46A

Fig. 2

#### Description.

Scales oval, 5.8–7.1 × 4.1–4.6 µm. A wide posterior rim (0.6–1.1 µm) encircles approximately one-half of the scale’s perimeter on its posterior part. The anterior part of the scale is bordered by a narrow, trough-shaped hyaline belt along a narrow, thickened edge. A thick secondary siliceous layer with an internal reticulation pattern of rounded mesh covers most of the scale surface, typically from two-thirds to four-fifths of it. This area contains numerous base plate pores and papillae on the surface of the secondary silica layer. In the proximal part of the scale, the secondary silica layer is significantly thinner and is absent around a large rimmed pore located centrally in this area. This central rimmed pore is surrounded by a “cloud” of small pores. Additional pores penetrate the entire proximal part of the scale, where the secondary siliceous layer is thinner. These pores are smaller in size than those in the distal part of the scale covered with the thick secondary layer.

Cysts and bristles were not observed.

#### Type.

Philippines • Mindanao Island, Bukidnon, Don Carlos, Barangay Pinamaloy, Lake Pinamaloy; 7°40.436'N, 125°00.063'E; 21 June 2025; E.S. Gusev leg. ***Holotype*.** Portion of a single gathering of cells on SEM stub 70_I_1, deposited at the Herbarium of the Steppe Institute of the Ural Branch of the Russian Academy of Sciences, Orenburg (ORIS). Fig. 2A is a representative scale from the type specimen.

#### Etymology.

Epithet “*pseudopustula*” is derived from the species *Mallomonas pustula* E.S. Gusev, Safronova, Podunay, Hoan Tran & Martynenko and Greek “pseudo” (= false).

#### Distribution and ecology.

In addition to the type locality, this species has been observed in the Fullbright Pond (Bukidnon, Maramag, Musuan, CMU Campus, 7°51.410'N, 125°2.998'E) and a water pool (Misamis Oriental, Gingoog City, Barangay Lunotan, 8°42.263'N, 125°00.923'E). This species has also been previously recorded in Vietnam, Dong Nai Province (Gusev et al. 2022a, fig. 4f, p. 210 as *Mallomonas* sp. 1). *Mallomonas pseudopustula* sp. nov. was found under the following environmental conditions: pH 6.6 to 7.3, specific conductance of 49 to 169 µS cm^−1^ and a temperature range of 28 to 31 °C (Table 1).

## ﻿Discussion

Our study revealed a relatively rich chrysophyte flora in the relatively small number of waterbodies investigated. In terms of species richness, the flora of Mindanao Island (36 taxa) is comparable to the known floras of silica-scaled chrysophytes of other tropical regions, such as Indonesia (45 taxa, Kapustin et al. (2019); Gusev et al. (2022b)), Madagascar (42 taxa, Hansen (1996a)), Malaysia (40 taxa, Dürrschmidt and Croome (1985)), the tropical areas of Brazil (47 taxa, Couté and Franceschini (1998); Franceschini and Kristiansen (2004) etc.), Sri Lanka (29 taxa, Dürrschmidt and Cronberg (1989)) and more than in such Asian countries as Papua New Guinea (19 taxa, Vyverman and Cronberg (1993)), Bangladesh (14 taxa, Takahashi and Hayakawa (1979)) and Singapore (9 taxa, Neustupa and Řezáčová (2007)). A greater number of species is known from regions with more extensive research efforts, such as Vietnam (108 taxa, Gusev et al. (2023a) etc.), India (58 taxa, Saha and Wujek (1990); Wujek and Saha (1996)) and the tropical areas of China (58 taxa, Wei and Yuan (2001); Wei et al. (2014)). Since all reported taxa are new records for the flora of the Philippines, we provide brief comments on their distribution patterns, grouping them by sections or species complexes.

The most represented group in terms of the number of taxa was the *Mallomonas mangofera* species complex. *Mallomonas mangofera* K. Harris & D.E. Bradley was previously considered a cosmopolitan species (Kristiansen 2000). It was originally described from Great Britain without a designated type (Harris and Bradley 1960) and its description was later expanded to include a morphotype from Chile (Dürrschmidt 1983). A recent revision, however, has shown that the morphotypes from Great Britain and Chile represent distinct species (Gusev et al. 2024b, c). *Mallomonas mangoferasensu stricto* appears to be restricted to the temperate zone of Eurasia and Japan, while findings from other regions identified as *M. mangofera* represent different morphotypes and species (Gusev et al. 2024b, c). The morphotype described from Chile, *M. mangofera apud* Dürrschmidt (Dürrschmidt 1983), was found on Mindanao Island (Fig. 3T). This morphotype is widely distributed in the tropical region, but requires revision and formal description as a new species (Gusev et al. 2023a). One widely-distributed taxon from the *M. mangofera* complex, *M. foveata* (Dürrschmidt) E.S. Gusev, was recorded in the Philippines (Fig. 3F–H). Recently, a number of new species have been described from the Tropics, based on both morphological and molecular criteria. *Mallomonas cetosa* Sterlyagova, Ignatenko, Nguyen Thi Lan & E.S. Gusev (Fig. 3A) is a recently described species that is widespread in the tropics, where it had been misidentified for many years under various names, most commonly as M. mangofera var. reticulata (Sterlyagova et al. 2025). *Mallomonas fragariformis* Gusev, Kapustin, Shkurina & Martynenko (Fig. 3I) and *M. minuscula* Gusev, Guseva, Kezlya & Kulikovskiy (Fig. 4A, B) are other species recently described from Vietnam, based on scale ultrastructure, but with a more limited known distribution (Gusev et al. 2019a, 2022c, 2023a). *Mallomonas fragariformis*, besides Vietnam, is also known from Indonesia (Gusev et al. 2022c) and Australia (Němcová and Diaz-Pulido 2023), while the *M. minuscula* has recently been reported from Australia (Němcová and Diaz-Pulido 2023). *Mallomonas hippocrepica* E.S. Gusev, Martynenko, Hoan Tran & Podunay (Fig. 3P) and *M. kornevae* E.S. Gusev, Martynenko, Hoan Tran & Podunay (Fig. 3S) were also recently described, based on morphological and molecular data (Gusev et al. 2024c). The finding of *M. hippocrepica* (Fig. 3P) in the Philippines is its first record outside of Vietnam, where it is known from only two localities (Gusev et al. 2024c). *Mallomonas kornevae* (Fig. 3S) is widespread in Vietnam and has already been found in Florida (Knotek et al. 2025), suggesting it may have a broad distribution in the Tropical Region.

Another species similar in ultrastructure to the *Mallomonas mangofera* complex is *M. favosa* K.H. Nicholls (Fig. 3E), which has been considered cosmopolitan (Kristiansen 2000; Kristiansen and Preisig 2007). *Mallomonas favosa* was originally described from Canada (Nicholls 1984) and later reported from other parts of the world (Kristiansen and Preisig 2007). However, our unpublished data indicate that morphotypes of *M. favosa* from the temperate zone of Eurasia (Czechia) and Vietnam differ significantly in their SSU rDNA and ITS rDNA nucleotide sequences, indicating they are different species (Gusev et al. 2024d; Škaloud et al. 2025). The *M. favosa* morphotype found in the Tropics is distributed in the freshwaters of Vietnam (Gusev et al. 2023a), Malaysia (Dürrschmidt and Croome 1985), China (Wei et al. 2014), Papua New Guinea (Vyverman and Cronberg 1993) and Brazil (Couté and Franceschini 1998; Franceschini and Kristiansen 2004). Another species from this group, *Mallomonas gemina* (Fig. 3K, L), is widely distributed in tropical Asia (Gusev et al. 2024d).

Morphotypes similar to *Mallomonas matvienkoaesensu lato* are widely distributed in the Tropics. For a long time, *M. matvienkoae* B. Asmund & Kristiansen, like *M. mangofera*, was considered a cosmopolitan taxon (Kristiansen 2000; Kristiansen and Preisig 2007). However, initial molecular studies revealed the heterogeneity of this group, leading to the description of four new species (Jo et al. 2013). The typification of *M. matvienkoae* and subsequent distribution analysis have shown that this taxon has so far been recorded only in Europe, Russia and South Korea (Gusev et al. 2024a; Škaloud et al. 2025). Findings from other regions belong to different species, representing a complex of morphologically similar species (Gusev et al. 2024a).

Amongst the species of the *Mallomonas matvienkoae*/*pseudomatvienkoae* group, the following were recorded on Mindanao Island: *M. pseudomatvienkoae* Jo, Shin, Kim, Siver & Andersen (Fig. 4L, M), *M. pustula* E.S. Gusev, Safronova, Podunay, Hoan Tran & Martynenko (Fig. 4N), *M. kapustinii* E.S. Gusev, Safronova, Podunay, Hoan Tran & Martynenko (Fig. 3Q), *M. paragrandis* Gusev (Fig. 4G) and *M. pseudopustula* sp. nov. (Fig. 2). We also provide images of a putative new species, designated here as *Mallomonas* sp. 2 (Fig. 4R) and *Mallomonas* sp. 3 (Fig. 4S), which requires further study. They differ from the known species by the development of the secondary silica layer surrounding the rimmed pore on the proximal part of the scale.

*Mallomonas pseudomatvienkoae* is a widely distributed species. It was described from South Korea (Jo et al. 2013) and its presence has been confirmed by molecular data in Vietnam (Gusev et al. 2024e). Morphotypes of this taxon have also been reported from India (Saha and Wujek 1990), Indonesia (Kapustin and Gusev 2019), Australia (Němcová and Diaz-Pulido 2023), Russia (Gusev et al. 2019b), France (Němcová et al. 2012) and Czechia (Němcová 2010), although these records require molecular confirmation. *Mallomonas pustula* and *M. kapustinii* were described from waterbodies in Vietnam (Gusev et al. 2024e). Scales of *M. pustula* also reported from other tropical regions (Gusev et al. 2024e). The record of *M. kapustinii* from Mindanao Island is the first outside Vietnam. *Mallomonas paragrandis*, also described from Vietnamese waterbodies a decade ago, is a species widespread in the Tropics and is frequently reported in Asian freshwaters (Gusev et al. 2023a). Scales corresponding to *Mallomonas paragrandis* (Fig. 4G), as well as scales morphologically similar to *M. paragrandis*, but differing by their larger size (6.3–7.0 × 3.7–4.1 µm vs. 4.8–6.0 × 2.7–4.0 μm), were discovered in waterbodies on Mindanao Island. The latter were identified as Mallomonas aff. paragrandis (Fig. 4H). Further molecular studies are required to determine whether these scales represent a new, previously unknown taxon or fall within the morphological variability of *M. paragrandis*, a species that may require a revision and an expansion of its morphometric characterisation.

As a result of this study, a new species, *Mallomonas pseudopustula* sp. nov. (Fig. 2), was described. *M. pseudopustula* belongs to section Planae. In scale shape and ultrastructure, *M. pseudopustula* resembles taxa from the *M. pseudomatvienkoae* and *M. matvienkoae* species complexes. *Mallomonas pseudopustula* is most similar to *M. pustula*. The scales of both species are oval, encircled by a wide posterior rim in the posterior part and a thickened narrow rim in the anterior part. They possess a thick secondary siliceous layer with internal reticulation comprising rounded meshes, numerous small pores on the base plate and papillae on the surface in the distal part and a large, rimmed pore in the proximal part. The scales of the compared species are similar in size: 5.8–7.1 × 4.1–4.6 μm in *M. pseudopustula* vs. 5.3–6.8 × 3.3–4.4 μm in *M. pustula*. A minor difference is observed in the width of the posterior rim surrounding the scale’s posterior part; it is wider in *M. pseudopustula* (0.6–1.1 μm vs. 0.48–0.75 μm in *M. pustula*). However, the principal distinction between the species lies in the ultrastructure of the scale’s proximal part. In *M. pseudopustula*, the large, rimmed pore located in the proximal part is surrounded by a cluster of numerous small pores; similar pores penetrate the entire proximal area of the scale. In contrast, the proximal part of the scale in *M. pustula* lacks small pores and contains only a single large, rimmed pore.

The presence of small pores in the proximal part of the scale clearly distinguishes *Mallomonas pseudopustula* from *M. pseudomatvienkoae* and *M. kapustinii* (Jo et al. 2013; Gusev et al. 2024e). Beyond this character, there are several other differences. *M. pseudopustula* differs from *M. pseudomatvienkoae* in its larger scales (5.8–7.1 × 4.1–4.6 μm vs. 3.0–5.0 × 2.0–3.0 μm) and the presence of papillae in the distal part. The new species differs from *M. kapustinii* by the presence of numerous papillae in the distal part of the scale (Jo et al. 2013; Gusev et al. 2024e).

The large size of the scales, the presence of a secondary siliceous layer and papillae in the distal part and numerous pores in the proximal part indicate a similarity between *M. pseudopustula* and *M. lamii* Gusev, Kulizin, Guseva, Shkurina & Kulikovskiy, *M. pleuriforamen* Siver, Lott, Jo, Shin, Kim & Andersen and *M. sorohexareticulata* Jo, Shin, Kim, Siver & Andersen (Jo et al. 2013; Gusev et al. 2019d). *Mallomonas pseudopustula* differs from these species in the extent of development of the secondary siliceous layer. In *M. pseudopustula*, it covers two-thirds to four-fifths of the scale surface, whereas in the compared taxa, it covers only one-third. Furthermore, in all the aforementioned species, the pore located in the proximal part of the scale is surrounded by a smooth hyaline zone, while in *M. pseudopustula*, it is surrounded by an aggregation of small pores. In the fossil species *M. pleuriforamen*, there are several such large pores in the proximal part.

*Mallomonas pseudopustula* also shares similarities with *M. limbata* Safronova & Gusev and *M. loricata* Gusev, Shkurina & Kulikovskiy regarding the extent of development of the secondary siliceous layer, which covers most of the scale and the presence of small pores in the proximal part. However, it differs in scale shape and several other important characteristics (Gusev et al. 2021b; Safronova et al. 2024). The scales of *M. pseudopustula* are oval, whereas those of *M. limbata* are ovoid, narrowed distally and those of *M. loricata* are obovate (Gusev et al. 2021b; Safronova et al. 2024). The scales of *M. pseudopustula* are larger (5.8–7.1 × 4.1–4.6 μm) than those of *M. limbata* (4.0–5.1 × 2.4–3.2 μm) and comparable in size to those of *M. loricata* (5.7–6.8 × 3.8–4.4 μm). In *M. pseudopustula*, the base plate pores are distributed over the entire scale surface, including the distal part, while in *M. loricata*, pores are absent in the distal quarter. Additionally, on the scale surface of *M. loricata*, papillae are sparse or most often absent, whereas *M. pseudopustula* has numerous papillae covering most of the scale surface.

*Mallomonas pseudopustula* differs from *M. paragrandis* Gusev in scale shape, larger scale size (5.8–7.1 × 4.1–4.6 μm vs. 4.8–6.0 × 2.7–4.0 μm), as well as the size and arrangement of pores. In *M. paragrandis*, there are two types of pores in the proximal part – small ones surrounding the central large pore and pores of larger diameter located in the remaining area (Gusev 2015). Furthermore, in *M. paragrandis*, the pores are confined solely to the proximal part. In *M. pseudopustula*, both the area around the central pore and the entire proximal part of the scale are covered with relatively small pores, while the entire distal part with the thick secondary layer is covered with larger ones.

In its shape, scale size and pattern of pore arrangement, *Mallomonas pseudopustula* also differs from *M. okhapkinii* Martynenko, Shkurina & E.S. Gusev (Martynenko et al. 2024) and *M. matvienkoae* Asmund & Kristiansen emend. E.S. Gusev (Gusev et al. 2024a). The scales of *M. okhapkinii* are oval or ovoid and distally narrowed, while those of *M. matvienkoae* are oval or obovoid. Unlike *M. pseudopustula* (5.8–7.1 × 4.1–4.6 μm), the scales of *M. okhapkinii* (3.7–6.4 × 2.8–4.2 μm) and *M. matvienkoae* (3.0–5.0 × 1.7–3.6 µm) are smaller. The scales of *M. okhapkinii* and *M. matvienkoae* are characterised by a uniform arrangement of base plate pores of similar size and a lack of papillae on the scale surface.

*Mallomonas ceylanica* Dürrschmidt & G. Cronberg (Fig. 3B–D) and *M. peronoides* (K. Harris) Momeu & Péterfi (Fig. 4K) are classified within the section Planae Momeu & L.S. Péterfi (Kristiansen and Preisig 2007). They form a distinct clade in phylogenetic trees (Siver et al. 2015; Knotek et al. 2025), suggesting the potential need for establishing a separate section. Morphotypes within the *M. peronoides* group (*M. peronoides*, *M. bangladeshica* (E. Takahashi & T. Hayakawa) Siver & A.P. Wolfe, *M. stellata* G. Cronberg and *M. ceylanica*) exhibit transitional forms and possess unique additional structures on the scale surface, described as “flower-like” or “star-like”. The taxonomic significance of these structures remains questionable and phylogenetic relationships within the group are unclear due to a lack of nucleotide sequences in databases. Our unpublished data indicate that the number of species in this group exceeds the number of currently-described taxa. *Mallomonas ceylanica*, a relatively rare species, has been recorded from Sri Lanka (Cronberg 1989), China (Wei et al. 2014), India (Saha and Wujek 1990; Wujek and Saha 1996), South Korea (Siver et al. 2015) and Vietnam (Gusev et al. 2023a). It can be reliably identified, based on morphological characteristics. In contrast, *M. peronoides*, which is considered a widely-distributed species, comprises several morphotypes. This taxon requires a comprehensive revision incorporating molecular methods.

Species of the section Papillosae Asmund & Kristiansen are widely distributed in the Tropics, with more than half of its species having been described from this region (Wujek 1984; Dürrschmidt and Croome 1985; Gusev 2013; Gusev et al. 2016, 2018, 2023c). In the Philippines, we recorded both widely distributed taxa and species with restricted distributions. A new morphotype requiring further study was also observed. *Mallomonas rasilis* Dürrschmidt (Fig. 4P, Q) can be considered a widely-distributed taxon, reported from both tropical and temperate zones. However, several distinct morphotypes and variations in bristle structure have been documented for this taxon, suggesting the presence of hidden diversity (Gusev et al. 2018). In contrast, *M. furtiva* Gusev, Certnerová, Škaloudová & Škaloud (Fig. 3J) has a distribution restricted to the Tropics. Besides Vietnam, where it was described, it is also known from Indonesia (Gusev et al. 2022b) and Malaysia (Dürrschmidt and Croome 1985). *Mallomonas tropica* Dürrschmidt & Croome (Fig. 4O) is a very rare taxon originally described from Malaysia (Dürrschmidt and Croome 1985) and recently found in eight localities in Vietnam (Gusev 2013; Gusev et al. 2022d; Gusev and Martynenko 2022). The Philippines represent the third known region for this species. Another group within the section Papillosae comprises morphotypes which are ultrastructurally similar to *M. guttatasensu lato.* Three morphotypes were found on Mindanao Island: M. guttata var. guttata Wujek (Fig. 3M), M. guttata var. simplex Nicholls (Fig. 3N) and a new morphotype designated here as *Mallomonas* sp. 1 (Fig. 3O).

Mallomonas guttata var. guttata was described from Costa Rica (Wujek 1984) and has been reported from numerous tropical countries, including Bangladesh (Takahashi and Hayakawa 1979), Indonesia (Cronberg and Hickel 1985, as *M. perforata* Cronberg and Hickel), Malaysia (Dürrschmidt and Croome 1985), Sri Lanka (Dürrschmidt and Cronberg 1989), Jamaica (Cronberg 1989), Zimbabwe (Cronberg 1989), China (Wei and Yuan 2001; Wei et al. 2014), Papua New Guinea (Vyverman and Cronberg 1993), India (Saha and Wujek 1990; Wujek and Saha 1996), Madagascar (Hansen 1996a), Nigeria (Wujek et al. 2003, 2010), Brazil (Couté and Franceschini 1998; Franceschini and Kristiansen 2004), Colombia (Vigna et al. 2005) and Ecuador (Wujek and Dziedzic 2005). However, morphological variations have been observed amongst specimens identified as M. guttata var. guttata, particularly in the arrangement, structure and number of pits on the shield (Gusev et al. 2017, 2021a). This suggests it may represent a complex of closely-related species, requiring further molecular studies. Mallomonas guttata var. simplex was described from Canada (Nicholls 1989). It is a doubtful taxon, differing from the typical M. guttata var. guttata by having fewer pits on the central area, arranged in a single row. Some studies have shown cells with scales exhibiting both multiple and single rows of pits (Gusev et al. 2017), indicating a need for detailed revision of this taxon. The new morphotype, *Mallomonas* sp. 1 (Fig. 3O), is clearly distinguished from M. guttata var. guttata by the presence of ring-shaped thickenings around the pits. We propose that this morphotype represents a new species, as it forms a distinct clade in our phylogenetic analysis (unpublished data). Its taxonomic status will be clarified following further investigation.

*Mallomonas parvula* Dürrschmidt (Fig. 4I) from the section Ouradiotae Asmund & Kristiansen, is a common species in temperate latitudes (Kristiansen and Preisig 2007). It is rare in the Tropical Region, with records from China (Wei and Yuan 2001), Nigeria (Wujek et al. 2003, 2010), Ecuador (Wujek and Dziedzic 2005) and a single finding from northern Vietnam (Gusev et al. 2021a). According to recent molecular data, two distinct genetic lineages (species) corresponding to this morphotype are known in culture from European waterbodies (Škaloud et al. 2025). Thus, molecular data are essential for revising this taxon and clarifying the taxonomic status of its tropical records.

*Mallomonas multisetigera* Dürrschmidt (Fig. 4F) is considered a cosmopolitan taxon (Kristiansen 2000) and represents a group with other species from the section Multisetigerae Asmund & Kristiansen, which is characterised by features reminiscent of fossil taxa (Gusev and Siver 2016). In the Tropical Region, it has been recorded from Jamaica (Cronberg 1989), China (Wei et al. 2014), Madagascar (Hansen 1996a), Nigeria (Wujek et al. 2003, 2010), Brazil (Wujek and De Bicudo 1993; Franceschini and Kristiansen 2004), Indonesia (Gusev et al. 2022b), Vietnam (Gusev et al. 2023a) and Ecuador (Wujek and Dziedzic 2005). It has also been reported from Europe (Škaloud et al. 2013b).

*Mallomonas morrisonensis* Croome & P.A. Tyler (Fig. 4C–E, J) and *M. kenyana* (Wujek & Asmund) D. Kapustin & E.S. Gusev (Fig. 3R) belong to the section Mallomonas Asmund & Kristiansen and are widely distributed species in the Tropical Region. *Mallomonas morrisonensis* was described from Australian waterbodies (Croome and Tyler 1983). It has been recorded in Malaysia (Dürrschmidt and Croome 1985), China (Wei and Yuan 2001), India (Saha and Wujek 1990; Wujek and Saha 1996), Madagascar (Hansen 1996a), Brazil (Franceschini and Kristiansen 2004) and Vietnam (Gusev et al. 2023a). *Mallomonas morrisonensis* was recorded in three waterbodies on Mindanao Island (Table 2). In one of them, alongside scales of typical morphology, domeless body scales with a diagonal rib arrangement on the shield were observed (Fig. 4E). Similar alterations in the form of aberrant or deviating rib patterns have been reported for *Mallomonas striata* Asmund, found in two main tributaries of Lake Baikal (Bessudova et al. 2018). Several authors associate the occurrence of such morphological changes with variations in key environmental factors. For instance, Němcová and Pichrtová (2012) documented variability in scale shape along a pH gradient in *M. striata*. Furthermore, changes in scale size and bristle length in response to increased cultivation temperature are known for *Mallomonas tonsurata* Teiling, *Mallomonas kalinae* Řezáčová, *Synura petersenii* Korshikov and *Synura curtispina* (J.B.Petersen & J.B.Hansen) Asmund (Gutowski 1996; Řezáčová-Škaloudová et al. 2010; Pichrtová and Němcová 2011). Therefore, it can be suggested that the presence of atypical scales in *M. morrisonensis* also represents a cellular response to changes in environmental conditions. *Mallomonas kenyana* was initially described as a variety, M. cyathelata Wujek & Asmund var. kenyana (Wujek and Asmund 1979) and was later elevated to species rank (Kapustin and Gusev 2019). It is a predominantly tropical species, known from the subtropics of the USA (Florida; Wujek (1984)), Kenya (Wujek and Asmund 1979), Zimbabwe (Cronberg 1989), Guatemala (Cronberg 1989), China (Wei and Yuan 2001), Papua New Guinea (Vyverman and Cronberg 1993), India (Saha and Wujek 1990; Wujek and Saha 1996), Madagascar (Hansen 1996a), Nigeria (Wujek et al. 2003, 2010), Colombia (Vigna et al. 2005), Vietnam (Gusev et al. 2023a) and Brazil (Franceschini et al. 1996).

*Synura longitubularis* B.Y. Jo, W. Shin, J.I. Kim & P. Siver (Fig. 4T) was recorded in several waterbodies of Mindanao Island. This is a relatively recently-described taxon from section Curtispinae Jo, Kim, Shin, Škaloud & Siver and is morphologically similar to *S. curtispina* (Jo et al. 2016). Information on the distribution of *S. longitubularis* is currently scarce. The species was first reported in Korea (Jo et al. 2016) and has only recently been discovered in China (Hao et al. 2022), Vietnam (Gusev et al. 2023a) and Australia (Němcová and Diaz-Pulido 2023). The Philippines is only the fifth region where *S. longitubularis* has been recorded. However, due to the minor morphological differences between *S. longitubularis* and *S. curtispina*, molecular confirmation of its record in the Philippines is required.

Two representatives of the genus *Spiniferomonas* were found in Mindanao Island: the cosmopolitan taxon *Spiniferomonas trioralis* E. Takahashi (Fig. 5A, B) and the widely-distributed species S. cf. bourrellyi E. Takahashi (Fig. 5E). Records of *S. trioralis* and *S. bourrellyi* in Tropical and Subtropical Regions are quite numerous. These species have been reported from Chile (Dürrschmidt 1980), Brazil (Franceschini and Kristiansen 2004), Florida, Panama, Bangladesh, Malaysia, Sri Lanka (Cronberg 1989), Madagascar Island (Hansen 1996a), the Caribbean Island of Dominica (Wujek et al. 2010), Vietnam (Gusev et al. 2019c; Gusev and Martynenko 2022) and Australia (Croome and Tyler 1985, 1988; Němcová and Diaz-Pulido 2023).

*Lepidochromonas eiffelii* (H.A. Thomsen) Kapustin & Guiry is a widely-distributed species (Kristiansen 2000). It has been recorded in arctic, temperate, subtropical and tropical climates. However, despite its wide distribution, many researchers note that the species is rare in samples (Gogorev et al. 2018). On Mindanao Island, only a single tower-shaped scale of *L. eiffelii* (Fig. 5F) was found in one water body.

Three taxa of the genus *Paraphysomonas* were found in the waterbodies of Mindanao Island: P. cf. corynephora Preisig & D.J. Hibberd (Fig. 5G), P. uniformis subsp. hemiradia Scoble & Cavalier-Smith (Fig. 5C) and P. vulgaris subsp. vulgaris Scoble & Cavalier-Smith (Fig. 5D, H). Amongst them, P. vulgaris subsp. vulgaris had the highest frequency of occurrence and was found in half of the studied waterbodies. These three taxa should apparently be considered widely distributed, as their records span from arctic (Bessudova et al. 2022) to tropical latitudes (Gusev et al. 2022b; Němcová and Diaz-Pulido 2023). As studies by Scoble and Cavalier-Smith (2014) have shown, the identification of *Paraphysomonas*, based on morphological characteristics, does not always reflect the true picture of the species diversity within this group. A complete and correct description of *Paraphysomonas* requires molecular studies. It is likely that future research on the flora of silica-scaled chrysophytes of Mindanao Island using DNA metabarcoding will supplement the data on the diversity of *Paraphysomonas* in this region.

Thus, the flora of silica-scaled chrysophytes of Mindanao Island comprises numerous noteworthy records and composed predominantly of species with tropical distributions, including taxa with very few known localities. Species composition shows the greatest similarity to nearby regions, such as Malaysia, Indonesia and Vietnam. Our study documented two taxa recently described from Vietnam and recorded here for the first time outside that country, described one new species and identified three morphotypes requiring further detailed investigation. Further research in the Philippines will undoubtedly reveal a richer flora and novel species of chrysophytes.

## Supplementary Material

XML Treatment for Mallomonas pseudopustula

## ﻿Additional information

### Use of AI

No use of AI was reported.

### Author ORCIDs

Evgeniy S. Gusev https://orcid.org/0000-0002-7397-5808

Marina E. Ignatenko https://orcid.org/0000-0002-4451-7816

Chris Rey Lituanas https://orcid.org/0000-0002-8584-3757

Kyla Rose Gumapac https://orcid.org/0009-0008-8147-5820

Nikita A. Martynenko https://orcid.org/0000-0002-7286-003X

Boris D. Efeykin https://orcid.org/0000-0003-1153-9995

### Data availability

All of the data that support the findings of this study are available in the main text.
